# Regulation of Antibiotic Use in Livestock: European and International Strategies to Prevent and Control Antimicrobial Resistance and Ensure Animal Welfare

**DOI:** 10.3390/antibiotics15010067

**Published:** 2026-01-08

**Authors:** Michela Maria Dimuccio, Virginia Conforti, Francesco Emanuele Celentano, Elena Circella, Anna Salvaggiulo, Giancarlo Bozzo, Marialaura Corrente

**Affiliations:** Department of Veterinary Medicine, University of Bari Aldo Moro, Strada Provinciale per Casamassima, km 3, 70010 Valenzano, Italy; michela.dimuccio@uniba.it (M.M.D.); v.conforti@phd.uniba.it (V.C.); francesco.celentano@uniba.it (F.E.C.); elena.circella@uniba.it (E.C.); anna.salvaggiulo@uniba.it (A.S.); marialaura.corrente@uniba.it (M.C.)

**Keywords:** antimicrobial resistance, antimicrobial use, livestock, One Health

## Abstract

Antimicrobial resistance (AMR) represents a significant global concern, undermining the efficacy of treatments in both human and veterinary medicine. Livestock production plays a major role in the emergence and dissemination of AMR, primarily due to the extensive use of antibiotics for therapeutic, prophylactic, and metaphylactic purposes. Addressing this multifaceted issue necessitates a One Health approach. At the international level, regulatory frameworks are predominantly non-binding, relying on soft-law instruments developed by the World Health Organization (WHO), the Food and Agriculture Organization (FAO), and the World Organization for Animal Health (WOAH, formerly OIE), which advocate for harmonized guidelines and national action plans. In contrast, the European Union has implemented binding regulations, including Regulation (EU) 2019/6 and Regulation (EU) 2019/4, which restrict non-essential antimicrobial use (AMU) and reinforce veterinary accountability. Initiatives such as the Farm to Fork Strategy and platforms like ClassyFarm further advance antimicrobial stewardship by integrating animal welfare, sustainability, and access to EU funding. Achieving substantial reductions in AMR within livestock systems requires coordinated, cross-disciplinary, and multi-level governance efforts. The EU model illustrates how enforceable legal frameworks, combined with science-based monitoring and welfare incentives, can facilitate prudent antibiotic use and promote sustainable animal production. This review aims to provide an integrated overview of international and European strategies for regulating antibiotic use in food-producing animals, focusing on how scientific, veterinary and legal perspectives contribute to combating AMR and promoting animal welfare by emphasizing prevention, and a prudent and responsible AMU.

## 1. Introduction

The discovery of antibiotics in the twentieth century represented a pivotal advancement in the treatment and prevention of infectious diseases, leading to a substantial reduction in bacterial infection mortality rates. Described as “magic bullets” by Erlich, antibiotics have profoundly influenced both medicine and society, notably improving life expectancy and quality since World War II, alongside advancements in nutrition and hygiene [1]. For instance, Armstrong et al. (1999) documented a marked decline in infectious disease mortality in the United States of America (USA) during the twentieth century, particularly coinciding with the introduction of antibiotic therapies between the 1920s and 1960s [2].

In fact, most antibiotics were discovered during the so-called Golden Age, a period from the 1940s to the 1970s [3].

Notably, the most significant advancements in antibiotic research occurred when pharmaceutical companies operated on a smaller scale and scientific knowledge was less advanced than today. Concurrent with the discovery of antibiotics, the phenomenon of antimicrobial resistance (AMR) was already being observed. In 1945, Alexander Fleming, during his Nobel Prize acceptance speech, cautioned about the potential emergence of AMR [4].

The inappropriate use of antibiotics in various settings has contributed to the development of AMR. Addressing AMR is a complex challenge that must be prioritized by both public health and veterinary sectors. AMR bacteria and associated resistance genes are commonly found in livestock, particularly within the gastrointestinal tract. Multidrug-resistant (MDR) bacteria, including Extended Spectrum Beta-Lactamase (ESBL)-producing enterobacteria and Methicillin-resistant *Staphylococcus aureus* (MRSA), are frequently identified in both animals and humans [5].

Addressing this issue necessitates a One Health approach, involving collaboration among physicians, veterinarians, and legal professionals. While AMR is frequently examined from clinical or microbiological perspectives, legal frameworks also play a critical role. Regulation determines the use of antimicrobials in animals, delineates professional responsibilities, and establishes parameters for prevention and control [6].

In livestock production, antibiotics are administered not only for therapeutic purposes but also for infection prevention and, in certain regions, for growth promotion. Without appropriate regulation, these practices can facilitate the emergence of resistant bacterial strains, posing risks to both animal and human health [7].

Legal frameworks are essential for a coordinated response to AMR. In recent years, regulatory initiatives have increasingly embodied the One Health approach, which acknowledges the interdependence of human, animal, and environmental health [6,8]. This shift has fostered greater alignment between public health objectives and veterinary practices, underpinned by international guidelines [9,10] and national legislation [11]. At both the European Union (EU) and international levels, legal instruments have been employed to restrict non-essential antimicrobial use (AMU), enhance surveillance, and promote responsible veterinary oversight. Nevertheless, effective legal frameworks must extend beyond prohibition, supporting animal welfare by ensuring access to necessary treatments and adapting to scientific advancements and farming systems.

Therefore, this review aims to provide an integrated overview of international and European strategies for regulating antibiotic use in food-producing animals, focusing on how scientific, veterinary and legal perspectives contribute to combating AMR and promoting animal welfare by emphasizing prevention and a prudent and responsible AMU. Moreover, this paper highlights how coordinated, cross-sectoral actions, grounded in the One Health approach, may support the long-term sustainability of animal production systems.

## 2. AMR: Anthropogenic Effects on a Natural Phenomenon

AMR is a natural phenomenon by which bacteria are able to adapt to environmental situations and selective pressure to which they are subjected.

Accordingly, the phenomenon of AMR is not unexpected, as many antibiotics are produced by bacteria. These molecules contribute to homeostasis and competition among bacterial species, with the corresponding genes embedded in the bacterial genome [12]. Multiple studies have documented the isolation of MDR bacteria in aboriginal populations [13] and, in veterinary contexts, in wild animals [14,15]. In both cases, MDR bacteria were found in individuals presumably never exposed to antibiotic therapy. These bacteria may also exhibit resistance to synthetic molecules, indicating that related genes existed in the pre-antibiotic era but likely served different biological functions [16].

However, the widespread use and misuse of antibiotics across medicine, veterinary medicine, and agriculture has accelerated the spread of AMR to alarming levels. AMR bacteria, and especially MDR bacteria or superbugs, significantly compromise the effectiveness of available treatments. The development of new active ingredients has not been sufficient to counteract AMR. Furthermore, the globalization of trade in people, animals, and food contributes to the dissemination of resistant bacteria, irrespective of local preventive measures [17].

MDR bacteria are frequently opportunistic species with large genomes, such as *Pseudomonas*, *Acinetobacter*, and *Stenotrophomonas*. The antibiotic era, which spans less than a century compared to the millions of years of the pre-antibiotic era, has imposed intense evolutionary pressure on bacteria. This has resulted in the proliferation of AMR bacterial clones, alterations in enzyme function, and the emergence of additional resistance mechanisms [16]. These developments underscore the profound impact of human antibiotic use over a relatively short period on a phenomenon that has existed for millennia [18,19].

AMR occurs by diffusion of mutations in the genome, events common to all living organisms, but also by gene transfer, a prerogative of prokaryotes. Looking at the history of antibiotic discovery/development, there is a definite synchrony between the use and appearance of AMR. Indeed, the more a drug is used, the faster bacteria exhibit resistance. For example, in the case of penicillin, AMR was evidenced even before it was used massively, during World War II. Vancomycin, being a drug not devoid of side effects, was employed less, and thus AMR arose at a later time [20]. Conversely, AMR against fluoroquinolones appeared very early, given the manageability of these antibiotics and their wide use [21].

### The Blame Game About AMR: One Health, One Fight

As awareness of AMR increased, the relevant professional groups—including physicians, veterinarians, and pharmacists—began to engage in collective reflection, though this process was often accompanied by mutual attribution of blame.

Regarding veterinary medicine, in 1969, the Swann Report was published, as a result of the work of a Committee appointed by the British government and consisting of representatives of various professions [22]. The lucid premises of that document, ahead of its time, highlighted the consequences of antibiotic treatments in various contexts. The Swann Report contained a claim of the One Health concept: “Bacterial populations should be regarded as a ‘unicum’ found in nature. The use of antibiotics in veterinary medicine has an impact, indirectly, on human health as well. Therefore, surveillance of antibiotic use, both in relation to animal and human welfare, appears crucial” [22].

## 3. The Supranational Legal Framework: Between Hard and Soft Law

Law—when integrated with science and policy—plays a pivotal role in building sustainable and resilient health systems, particularly in the face of global challenges such as AMR. In the specific context of livestock production, regulatory strategies must navigate between promoting animal health, ensuring food safety, and preserving antibiotic effectiveness.

At the international level, the legal response to AMR has largely relied on soft-law instruments—non-binding guidelines, action plans, and recommendations that influence national legislation and regulatory behaviours through consensus and coordination rather than enforcement. While these tools lack formal legal obligation, they play a fundamental role in shaping national agendas, guiding investments, and promoting harmonised practices across countries.

A central actor in this landscape is the Tripartite collaboration between the World Health Organization (WHO), the Food and Agriculture Organization (FAO), and the World Organisation for Animal Health (WOAH, formerly OIE). Their “Global Action Plan on Antimicrobial Resistance” (2015) urges countries to reduce non-therapeutic use of antibiotics in animals and improve regulatory oversight through multisectoral national action plans [8,23]. This document laid the groundwork for the global adoption of the One Health paradigm.

The Codex Alimentarius, jointly developed by FAO and WHO, adds further normative value. Its “Guidelines for Risk Analysis of Foodborne Antimicrobial Resistance” promote risk-based approaches to manage AMR in the food chain, particularly regarding prudent AMU and surveillance [9]. Though not enforceable, Codex standards are referenced under the World Trade Organization (WTO) framework and may influence trade-related disputes and policy convergence.

WOAH has also contributed decisively through its Terrestrial Animal Health Code, which provides technical recommendations on AMU, including bans on growth promotion and the necessity of veterinary oversight. These standards are widely respected, and while voluntary, they significantly shape both national legislation and bilateral trade discussions [24].

On the political level, the United Nations Political Declaration on AMR (2016) elevated AMR to a priority on the global health agenda. It called for more robust national regulations and pledged international support for capacity building and surveillance [25]. Similar commitments have emerged from Group of Seven (G7), Group of Twenty (G20), and WHO governing bodies.

Despite growing international alignment, key limitations persist. Binding obligations remain rare, implementation is uneven—especially between high-income and low-income countries—and enforcement mechanisms are weak or non-existent. This hinders the translation of global strategies into measurable outcomes.

Nonetheless, international law retains strategic importance. It fosters a shared vocabulary, minimum standards, and common objectives. As scientific and political consensus around AMR deepens, these instruments may pave the way for future binding agreements—especially if anchored in the One Health framework and supported by legal capacity building.

### The Essential Role of European Union Regulatory Activity

In contrast to the international approach, largely based on soft law and voluntary adherence, the EU has developed a binding and highly integrated legal framework to address AMR in the veterinary sector. This framework directly shapes Member States’ legislation and practices, ensuring greater uniformity and accountability in the use of antimicrobials in livestock production.

At the core of this system lies Regulation (EU) 2019/6 on veterinary medicinal products, which entered into force in January 2022 [11]. This regulation imposes strict limitations on the use of antibiotics in animals: growth promoters (GPs) are explicitly banned, prophylactic use is permitted only under exceptional conditions, and metaphylactic use requires clear veterinary justification [11]. It also standardises procedures for marketing authorisations, strengthens pharmacosurveillance, and mandates data collection on AMU across Member States.

Complementing this, Regulation (EU) 2019/4 on medicated feed reinforces the principles of prudent antibiotic use by requiring veterinary prescriptions and regulating medicated feed production and distribution [26].

In parallel with these legal instruments, the European One Health Action Plan against AMR sets strategic priorities, such as improving surveillance systems and reducing antimicrobial sales [27]. These goals have been further expanded through the Farm to Fork Strategy (F2F)—a central component of the European Green Deal—which calls for a 50% reduction in antimicrobial sales for farmed animals and aquaculture by 2030 [28].

To operationalise these targets, the EU links antibiotic stewardship with broader sustainability incentives. The Common Agricultural Policy (CAP) now integrates AMR reduction into its funding mechanisms. For instance, the Italian CAP Strategic Plan makes access to subsidies conditional on participation in systems such as ClassyFarm, which monitors risk levels and antibiotic consumption on farms.

A key role in this ecosystem is played by the European Food Safety Authority (EFSA), which—together with European Medicines Agency (EMA) and European Centre for Disease Prevention and Control (ECDC)—publishes annual reports on AMR trends in zoonotic and indicator bacteria, offering essential data for policy updates and national implementation [29].

What sets the EU model apart is its regulatory enforceability, multi-level governance, and integration with sustainability goals. While heterogeneity in implementation remains a challenge—due to differences in veterinary infrastructure and prescribing habits—the overall structure ensures greater coherence than what is typically achievable through soft-law mechanisms.

Importantly, the EU’s regulatory model may serve as a reference point for other regions, such as the African Union or Mercado Común del Sur (MERCOSUR). However, the full exportability of this model may be limited by disparities in institutional capacity, surveillance infrastructure, and veterinary services. This underlines the need for context-specific adaptations if similar frameworks are to be implemented globally.

## 4. Growth Promoters, the Original Sin of Vets

Historically, antibiotics were administered orally at subtherapeutic dosages to enhance the productive performance of farm animals. The nutritional use of antibiotics, subsequently termed GPs, gained prominence following the incidental discovery that animals exhibited improved growth when fed with tetracycline-producing Streptomyces-contaminated feed [14]. As a result, GPs became common in the diets of chickens, pigs, and beef cattle [30].

From an animal husbandry standpoint, the use of GPs was initially justified, likely due to their modulatory effects on the gut microbiota. However, this practice predictably contributed to the proliferation of AMR. Notably, GPs could be administered without veterinary prescription. The Swann Report highlighted that administering antibiotics at subtherapeutic doses accelerates the development of AMR and recommended restricting such use to compounds not employed in human medicine [22]. Nevertheless, cross-resistance mechanisms, where exposure to one antibiotic facilitates resistance to others within the same or related classes, render the use of GPs problematic. For example, avoparcin use in poultry led to cross-resistance with vancomycin and the emergence of vancomycin-resistant enterococci (VREs). Similar mechanisms have been observed with other antibiotics, such as tylosin.

In certain Northern European countries, such as Sweden, GPs were prohibited as early as 1986. Sweden’s accession to the EU and the subsequent need for legislative harmonization facilitated the extension of this ban across the European Union [31]. Consequently, the EU implemented a comprehensive ban on GPs in 2006, followed by the USA in 2017 [32,33]. Currently, the use of GPs has markedly declined, even in countries where formal bans are not yet in place [34]. Therefore, it can be optimistically considered that the problem of GPs is mainly related to the past.

### 4.1. Metaphylaxis and Chemoprophylaxis: The Paradox of Treating Healthy Animals

As a flip side of the coin, despite the ban on GPs, antibiotics can be used on farms, at therapeutic dosages, for mass treatment and for preventive purposes. So-called metaphylaxis is the treatment of animals in the event of an outbreak of infectious disease with a bacterial etiology to stop the contagion while chemoprophylaxis is the antibiotic treatment at critical stages of farming, such as weaning or dry, to prevent infection. In both cases the use is not strictly justified as healthy animals are treated. That’s why Regulation (EU) 2019/6 of the European Parliament, in force since 2022, has regulated the use of such treatments, claiming that they should be considered an exception, rather than the rule [11], and emphasizing the centrality of the veterinarian, in the decision-making process. In Italy, the Legislative Decree of 7 December 2023 on Veterinary Medicines emphasizes that metaphylaxis should be carried out on the basis of a diagnostic assessment, isolating the bacteria responsible for the infection and testing them for the in vitro susceptibility to antibiotics [35].

### 4.2. Antibiotics as Magic Bullets, Between Classifications and Bans

Starting from the observation that the more an antibiotic is used, the faster bacteria develop AMR and remembering that use in any context, whether medical, veterinary, or agricultural, affects others, the scientific community has focused attention and on antibiotics of primary importance to human health. The WHO classification, now in its seventh revision in 2024, classifies antibiotics into three Medically Important antimicrobials (MIAs) categories [36]. This classification was made in consultation with the FAO, the United Nations Environmental Programme (UNEP), and the WOAH, with the goal of limiting the use of antibiotics important to human health as much as possible. In the WHO document, antibiotics are classified into three categories: (i) highest priority critically important; (ii) highly important; (iii) important.

Over time, the importance of some antibiotics has changed. For example, colistin, which was formerly an antibiotic of predominantly veterinary use, has become a last resort drug for the treatment of MDR bacterial infections, and currently is considered a critically important drug for human health, such as fourth-generation cephalosporins and fluoroquinolones [36].

**Figure 1:**
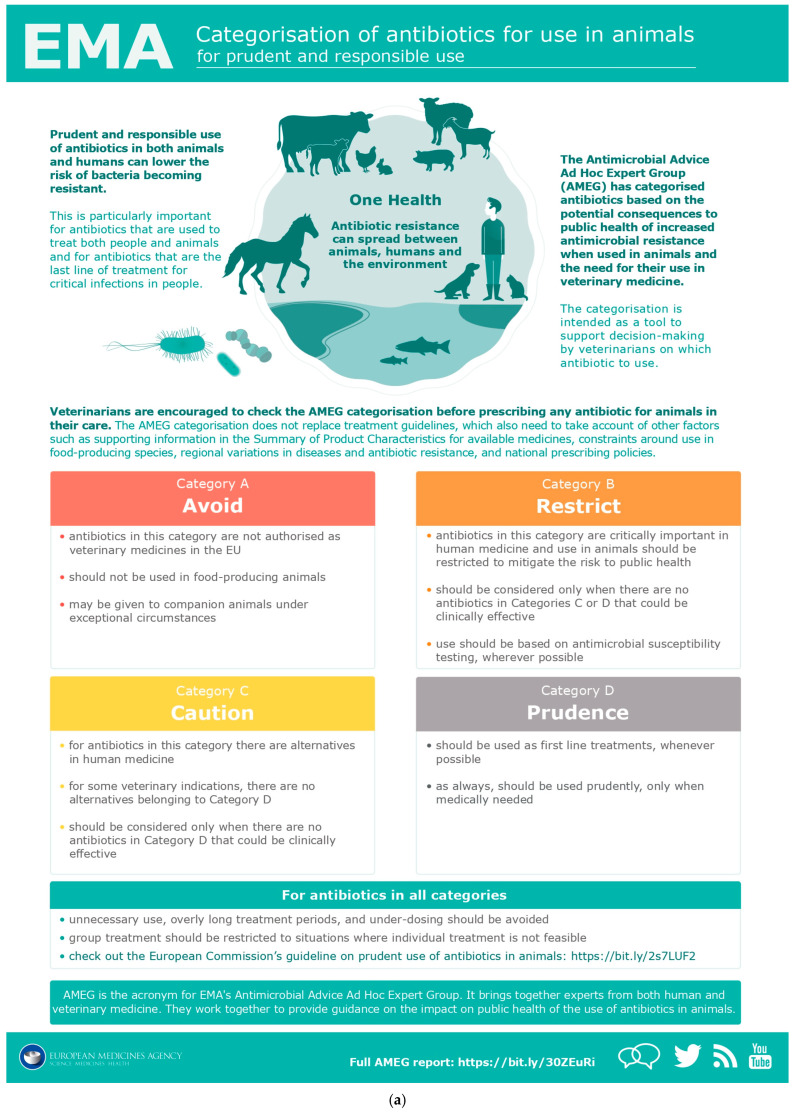

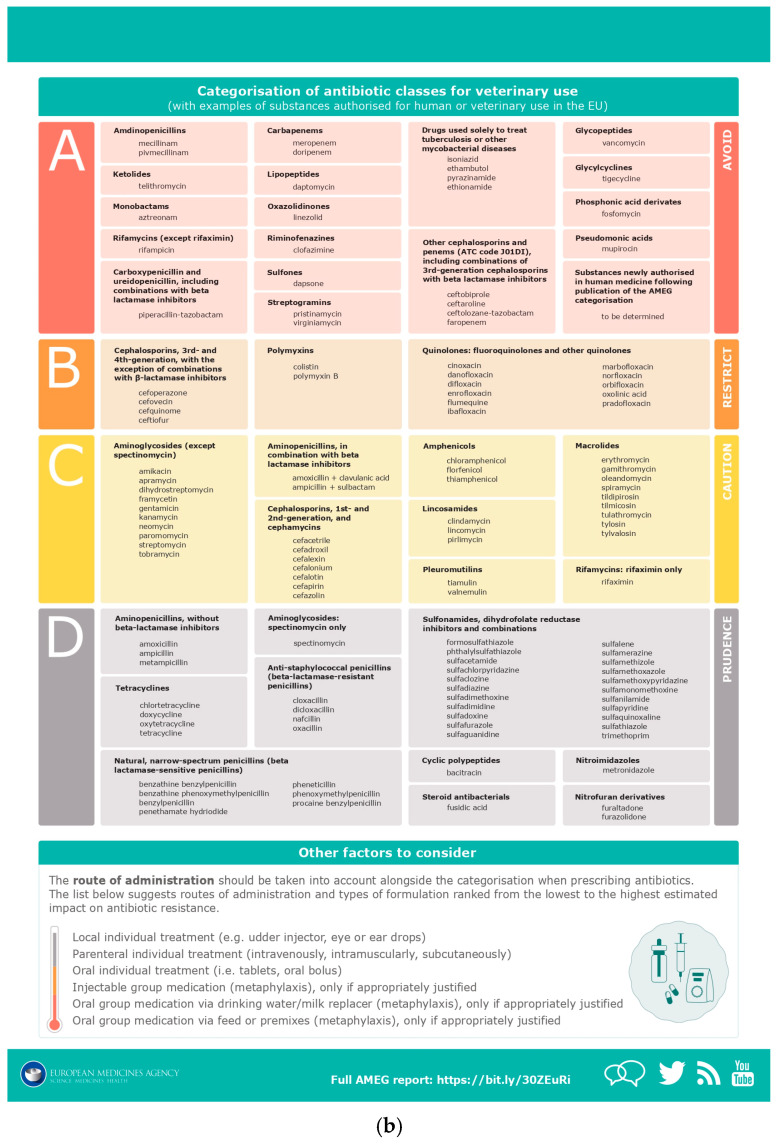
(**a**) EMA infographics on the categorization of antibiotics for use in animals [37]. (**b**) EMA infographics on the categorization of antibiotics for use in animals [37].

In line with the WHO categorization and the MIA list, the EMA has developed guidelines on the use of antibiotics in veterinary medicine, which is useful for the choice of active ingredients, especially whether first or second choice (Figure 1a,b).

This document in turn refers to categorization, made by the Antimicrobial Advice Ad Hoc Expert Group (AMEG) of the EMA [37].

Veterinary physicians are urged to consult AMEG categorization before prescribing antibiotics to animals in their care, especially if they are farm animals, considering the effect that the possible development of AMR due to their use in animals may have on public health.

In addition, in line with the guiding principle of the WHO list, European Regulation 2022/1955 banned in farm and companion animals, the use of certain antibiotics of critical importance to human health, such as carbapenems, glycopeptides and linezolid [38].

### 4.3. Rehabilitating the Figure of the Veterinarian

The misuse that has been made of antibiotics as GPs has generated a public perception that antibiotics are used incorrectly, so they should no longer be used in farm animals [39]. Although GPs have been banned for almost two decades, according to a 2022 Eurobarometer survey on the use of and knowledge about antibiotics by European citizens, about 3 in 5 respondents are unaware of the existence of the ban [15]. The feeling that antibiotics are used freely leads to the idea that these drugs should never be used in farm animals. In fact, when asked, “Do you think that farm animals should be treated with antibiotics when they are sick?”, about one-third of citizens surveyed responded that they disagreed, while 5 percent had no opinion on the matter. Such opinions are in firm contrast to animal welfare laws, and in evident contradiction to animal welfare consciousness [15]. Paradoxically, these feelings reach higher levels in a nation like Germany, where the environmentalist tradition is very deeply rooted (40 percent of German citizens reject the idea of treating sick farm animals) [15].

In addition to this, it is little known that there are elements of good practice which are established by law, related to the use of drugs in farm animals, antibiotics included:

(i) Maximum Residue Limit (MRL): This is the maximum allowed concentration of a residue in a food product obtained from an animal that has received a veterinary medicine or that has been exposed to a biocidal product for use in the animal husbandry sector. Before a veterinary medicine intended for food-producing animals is authorized in the EU, the Committee for Veterinary Medicinal Products (CVMP) evaluates the safety of the pharmacologically active substances in it and their residues and recommends MRLs accordingly. In this regard, the EMA has published scientific guidance relevant to the establishment of MRLs for veterinary medicines [40].
(ii) The withdrawal period: This is the period that must elapse between the last administration of the veterinary medicinal product to animals (under normal conditions of use and in accordance with the provisions of the Directive 2001/82/EC) and the production of foodstuffs from such animals. This in order to protect public health by ensuring that such foodstuffs do not contain residues in quantities exceeding the MRLs for active substances laid down pursuant to Regulation (EEC) No 2377/901 [11,41,42]. In line with article 12.3 of Directive 2001/82/EC, marketing authorization applications for veterinary medicinal products for use in food producing species must include an indication of the so-called withdrawal period [41].
(iii) Traceability centralized System of Veterinary Prescriptions. Veterinary electronic prescription (VEP) is mandatory by law since 2022 [11]. The adoption of a computer system for the traceability of veterinary medicinal products and medicated feed through the introduction of VEP is provided for pets and food producing animals. Any adverse drug reactions are also recorded in this traceability system [43,44,45].
(iv) Pharmacovigilance: This refers to all the operations aimed at identifying, assessing, understanding and preventing adverse effects or any other problems related to the use of medicinal products in both human and veterinary medicine. These activities aim to (1) increase knowledge about medicines and better define their safety, (2) to improve the way these molecules are used, (3) establish a safety profile that better corresponds to actual medical practice and (4) describe the characteristics of patients undergoing treatment in a more realistic manner [45]. The EU pharmacovigilance system, fed by the programmes of the medicines agencies of the various Member States (e.g., the Italian Medicines Agency (AIFA)), provides for the ongoing safety monitoring of medicines and their use through a continuous data collection on this aspect [45]. These data can be obtained from different sources: reports of suspected adverse reactions (spontaneous and non-spontaneous), clinical studies, scientific literature, reports submitted by pharmaceutical companies, etc. Such pharmacovigilance system also monitors: (i) Ineffectiveness or adverse events when a medicine is used off-label, (ii) adequacy of withdrawal periods, (iii) suspected spread of an infectious agent facilitated by a medicine, (iv) adverse effects on the environment [46]. With regard to the possible off-label use of medicines (for indications other than those officially authorized by EMA), public service veterinarians carry out checks at farms, clinics and veterinary practices [46,47].
(v) Pharmacosurveillance: It consists of monitoring activities throughout the entire drug supply chain (production, marketing, handling and administration) aimed at maintaining animal health and protecting consumers. This is a very important field of activity given its direct impact on animal welfare and safety of food of animal origin, e.g., in case of finding authorized or illegal drug residues. Pharmacosurveillance includes: (1) preventive ascertainments for granting permission for the possession of veterinary drug stocks in healthcare, detention and breeding facilities; (2) authorization procedures for the wholesale, direct sale and storage of veterinary drugs; (3) formal and substantial control of drug prescriptions; (4) sampling for the detection of illegal and/or authorized drug residues in different matrices. This kind of surveillance is carried out in various areas (on farms, in pharmacies and parapharmacies, wholesalers and direct retailers) annually and this requires planning of interventions of the competent authorities based on the risk analysis approach. The risk categorization of the facilities subject to control (primarily livestock farms) is currently carried out through information systems such as the ClassyFarm system [48,49].
(vi) ClassyFarm system: This is a voluntary subscription IT platform for categorizing livestock farms according to risk, which has been developed by the Italian Ministry of Health, in collaboration with the Istituto Zooprofilattico Sperimentale of Lombardia and Emilia Romagna (IZSLER) and the University of Parma, since 2017 [50,51,52,53,54,55]. ClassyFarm is the Italian response to the European Parliament’s call on Member States for new techniques and technologies for animal welfare, as part of the European Strategy for Animal Welfare 2016–2020 [56]. It is also part of the Italian strategy to combat AMR 2017–2020 (also known as “Piano Nazionale di Contrasto all’Antibiotico-Resistenza” (PNCAR) [57].

This system has been preceded by the Danish Integrated Antimicrobial Resistance Monitoring and Research Programme (DANMAP) and the Finnish Veterinary Antimicrobial Resistance Monitoring and Consumption of Antimicrobial Agents program (FINRES-Vet), which were fore runner AMR monitoring programmes set up in the wake of the Copenhagen Recommendations in 1998 [58,59,60]. The DANMAP, in more detail, was developed by the Danish Ministry of Food, Agriculture and Fisheries and the Danish Ministry of Health as the first European comprehensive database for surveillance of AMU and resistance in bacteria from food animals, food and humans. It has been enhanced in 2002 with the addition of the Veterinary Statistics Program (VETSTAT), which provides monthly sales data on medicinal products prescribed for veterinary consumption using pharmacies, veterinarians and feed mills as sources of information [61,62].

The FINRES-Vet, however, which has been in place since 2002, monitors the antibiotic sensitivity of zoonotic bacteria, animal pathogens, and indicator bacteria to provide data on the emergence of new resistance mechanisms in Finland. FINRES results and annual reports are consistently published and made publicly accessible on the Finnish Food Authority website [59].

In contrast to the aforementioned programs, ClassyFarm evaluates multiple aspects of each participating farm: (1) animal welfare; (2) biosecurity; (3) health and production parameters; (4) animal nutrition; (5) consumption of antimicrobial drugs; and (6) lesions detected at the slaughterhouse (e.g., lung lesions) [51,63]. Additionally, ClassyFarm can assess various livestock species, including swine, cattle, buffalo, sheep, goats, poultry, and rabbits, using specific checklists tailored to each aspect and subdivided according to the animals’ productive aptitude and the farming method employed [51].

The system manages a substantial internal data flow from various pre-existing sources, including the Italian Data Bank for Livestock Registration (BDN), the VEP, and diagnostic laboratories of the Istituti Zooprofilattici Sperimentali (IIZZSS), utilizing business intelligence processes. The platform also integrates data from official controls and self-monitoring activities conducted by Food Business Operators (FBOs) in collaboration with farm veterinarians [50,52]. This integration facilitates the identification of risks, defined as non-compliant situations according to ClassyFarm checklists, on each livestock farm and guides targeted interventions to minimize these risks [51,64]. Processed data are subsequently made available to registered users through interactive dashboards and downloadable reports [51].

In accordance with Reg. (EU) 2016/429, the ClassyFarm system aims to enhance the prevention of transmissible animal diseases, combat antimicrobial resistance, and improve the efficiency and targeting of official controls in terms of economic and human resources. Achieving these objectives requires the implementation of animal welfare and biosecurity measures on livestock farms. Several studies have demonstrated a significant reduction in AMU on dairy and beef cattle farms participating in ClassyFarm and adopting management plans informed by the system’s data [51,52,64,65,66].

Within this context, the number of farms participating in ClassyFarm in the Apulia Region increased substantially in the second half of 2023, coinciding with ClassyFarm becoming a prerequisite for accessing CAP Ecoscheme 1 rewards. CAP financial support serves as an incentive for farmers to avoid preventive treatments, such as metaphylaxis and chemoprophylaxis, and to adopt more sustainable farming practices.

Ensuring animal welfare does not entail prohibiting the use of antimicrobials, but rather promoting their appropriate use to safeguard the health of both animals and humans [67]. Prudent antibiotic use in animals is essential, as improper use increases the likelihood of antibiotic residues in food products such as milk, eggs, and meat. These residues can result in adverse effects, including the transfer of antibiotic-resistant bacteria to humans, immunopathological effects, allergies, mutagenicity, nephropathy, hepatotoxicity, reproductive disorders, bone marrow toxicity, and carcinogenicity.

Further investigation into the susceptibility of microorganisms present in the farm environment, in relation to antimicrobial consumption on ClassyFarm-participating farms, would be valuable and would align with practices established in the FINRES-Vet program. To support this objective, beginning in the second half of 2024, the ClassyFarm system has incorporated the “Stewardship and prescriptive appropriateness” functionality. This new dashboard enables the collection, management, and consultation of data on bacterial sensitivity to antimicrobials, based on samples from both food-producing and non-food-producing animals.

Pursuing antibiotic-free animal husbandry without regard for responsible use practices can be problematic. While antibiotic-free production is feasible in certain contexts, such as short production cycle farms like poultry farms, it remains unattainable in others. A 2019 survey in the USA found that breeders and veterinarians often view antibiotic-free farms as a marketing concept rather than a practical health standard [68]. Nevertheless, AMU has declined substantially at national, European, and global levels. Between 2010 and 2022, AMU in farm animals decreased by 53% [69]. The data are impressive not only for the quantity, but also for the category of antibiotics used: in 2022, the majority of antibiotics sold (79%) belonged to EMA category D, the most manageable. The data reported at the global level are also encouraging: a report of the WOAH highlights a 34% reduction in the consumption of antibiotics in the period 2015–2017 [34].

## 5. From Bans to Best Practices: How to Reduce the Use of Antibiotics

The evidence presented indicates that reducing AMU is best achieved through preventive rather than therapeutic strategies. Lowering the intensity of livestock farming can decrease animal stress and susceptibility to infectious diseases, although this may result in higher production costs. Effective communication is essential to ensure that consumers understand the relationship between increased expenditure and the safety of animal-derived products [70,71].

Subsequent to the Swann Report, additional forward-looking perspectives have emerged in the literature, though they have often been overlooked [22]. For example, Bernard Rollin, an American philosopher and bioethicist, questioned in 2001 whether the increased cost of meat resulting from bans on growth promoters or reduced intensification should be weighed against the potential costs of treating MDR bacterial infections [72].

Investing in the long run is needed [39,73]:

- Implementing external (prevention of pathogen entry into the farm) and internal (prevention of spread within the farm) biosecurity and hygiene standards, as the ClassyFarm system reiterates.
- Select disease-resistant genetic lines, such as by analyzing the microbiota of animals less susceptible to infectious diseases.
- Investing in vaccination programs.
- Improving animal nutrition beginning with good quality colostrum (in order to strengthen animals’ natural immunity).
- Providing adequate environmental conditions.
- Reinforcing all these conditions at times of increased animal stress.

Such processes can be time-consuming and expensive, but they are justified by the benefit to public health [39].

**Figure 2:**
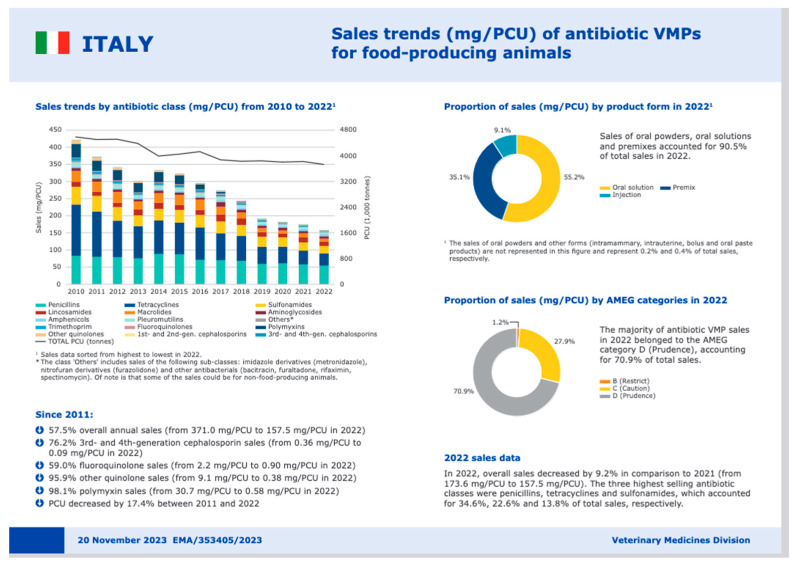
Sales trends of antibiotics used for food-producing animals from 2010 to 2022 in Italy [69].

When antibiotic treatment cannot be avoided, the support of laboratory analysis to identify the resistance profile of pathogens involved, as discussed above, is essential. Instead, in cases of empiric therapy, first-choice antibiotics should be used essentially, consulting the appropriate guidelines (Figure 2).

## 6. Conclusions: From Awareness to Action

AMR in livestock production exemplifies the tragedy of the commons: a shared resource—antibiotic efficacy—degraded by widespread misuse [74]. Beyond its clinical implications, AMR generates substantial economic burdens [75], raises ethical concerns by threatening the right to health [76], and contributes to environmental contamination through antibiotic residues [77].

This review has shown that effectively addressing AMR in animal production requires a coordinated, cross-sectoral strategy. Internationally, soft-law instruments—while limited in enforceability—have played a critical role in agenda-setting and normative convergence. At the European level, the integration of binding regulation, surveillance systems, and sustainability policies (e.g., F2F, CAP conditionalities) offers a governance model specifically designed to tackle AMR.

The review also highlighted the need to implement the following actions in the near future:

(i) Strengthen legal frameworks in non-EU regions through adoption of binding standards that regulate antibiotic use beyond growth promotion bans.
(ii) Promote animal welfare as a pillar of AMR reduction policies, recognizing its role in lowering disease incidence and antibiotic need.
(iii) Invest in farm-level data systems, such as ClassyFarm, to support risk-based decision-making, transparency, and accountability.
(iv) Support the role of veterinarians as gatekeepers of responsible use, through continuous education, diagnostic tools, and stewardship support.
(v) Engage consumers and policy-makers in recognizing that sustainable animal production may require trade-offs between cost, productivity and public health.

Solving controlling AMR is not just a technical challenge, but a governance test. A One Health approach must move from principle to implementation, integrating science, policy and law. These essential tools can only be preserved for future generations through shared responsibility and informed restraint—use antibiotics but less and better.

## Data Availability

No new data were created or analyzed in this study. Data sharing is not applicable to this article.

## Abbreviations

The following abbreviations are used in this manuscript:

USA	United States of America
AMR	Antimicrobial Resistance
MDR	Multidrug resistant
ESBL	Extended Spectrum Beta-Lactamase
MRSA	Methicillin-resistant Staphylococcus aureus
EU	European Union
AMU	Antimicrobial Use
WHO	World Health Organization
FAO	Food and Agriculture Organization
WOAH	World Organization for Animal Health
WTO	World Trade Organization
G7	Group of Seven
G20	Group of Twenty
GPs	Growth Promoters
F2F	Farm to Fork Strategy
CAP	Common Agricultural Policy
EFSA	European Food Safety Authority
EMA	European Medicines Agency
ECDC	European Centre for Disease Prevention and Control
MERCOSUR	Mercado Común del Sur
VREs	Vancomycin-Resistant Enterococci
MIAs	Medically Important antimicrobials
UNEP	United Nations Environmental Programme
AMEG	Antimicrobial Advice Ad Hoc Expert Group
MRL	Maximum Residue Limit
CVMP	Committee for Veterinary Medicinal Products
VEP	Veterinary Electronic Prescription
AIFA	Italian Medicines Agency
IZSLER	Istituto Zooprofilattico Sperimentale of Lombardia and Emilia Romagna
PNCAR	Piano Nazionale di Contrasto all’Antibiotico-Resistenza
DANMAP	Danish Integrated Antimicrobial Resistance Monitoring and Research Programme
FINRES-Vet	Finnish Veterinary Antimicrobial Resistance Monitoring and Consumption of Antimicrobial Agents program
VETSTAT	Veterinary Statistics Program
BDN	Italian Data Bank for Livestock Registration
IIZZSS	Istituti Zooprofilattici Sperimentali
FBOs	Food Business Operators

## References

[1] Zipfel P.F., Skerka C. (2022). From magic bullets to modern therapeutics: Paul Ehrlich, the German immunobiologist and physician coined the term ‘complement’. Mol. Immunol..

[2] Armstrong G.L., Conn L.A., Pinner R.W. (1999). Trends in infectious disease mortality in the United States during the 20th century. JAMA.

[3] Browne K., Chakraborty S., Chen R., Willco M.D., Black D.S., Walsh W.R., Kumar N. (2020). A New Era of Antibiotics: The Clinical Potential of Antimicrobial Peptides. Int. J. Mol. Sci..

[4] Fleming A. Nobel Lecture. Nobel Prize Outreach 2025. https://www.nobelprize.org/prizes/medicine/1945/fleming/lecture/.

[5] Ballash G.A., Parker E.M., Mollenkopf D.F., Wittum T.E. (2024). The One Health dissemination of antimicrobial resistance occurs in both natural and clinical environments. J. Am. Vet. Med. Assoc..

[6] McEwen S.A., Collignon P.J. (2018). Antimicrobial Resistance: A One Health Perspective. Microbiol. Spectr..

[7] O’Neill J. (2016). The Review on Antimicrobial Resistance. Tackling Drug-Resistant Infections Globally: Final Report and Recommendations.

[8] FAO, OIE, WHO, UNEP (2021). Antimicrobial Resistance and the United Nations Sustainable Development Cooperation Framework: Guidance for United Nations Country Teams.

[9] FAO, WHO (2023). Foodborne Antimicrobial Resistance—Compendium of Codex Standards.

[10] FAO, WHO (2023). General Principles of Food Hygiene. Codex Alimentarius Code of Practice, No. CXC 1-1969.

[11] The European Parliament and The Council of the European Union (2019). Regulation (EU) 2019/6 of the European Parliament and of the Council of 11 December 2018 on Veterinary Medicinal Products and Repealing Directive 2001/82/EC. O.J. L 4. https://eur-lex.europa.eu/eli/reg/2019/6/oj/eng.

[12] Martínez J.L. (2008). Antibiotics and antibiotic resistance genes in natural environments. Science.

[13] Clemente J.C., Pehrsson E.C., Blaser M.J., Sandhu K., Gao Z., Wang B., Magris M., Hidalgo G., Contreras M., Noya-Alarcón Ó. (2015). The microbiome of uncontacted Amerindians. Sci. Adv..

[14] Jukes T.H., Williams W.L. (1953). Nutritional effects of antibiotics. Pharmacol. Rev..

[15] Trotta A., Cirilli M., Marinaro M., Bosak S., Diakoudi G., Ciccarelli S., Paci S., Buonavoglia D., Corrente M. (2021). Detection of multi-drug resistance and AmpC β-lactamase/extended-spectrum β-lactamase genes in bacterial isolates of loggerhead sea turtles (*Caretta caretta*) from the Mediterranean Sea. Mar. Pollut. Bull..

[16] Aminov R.I. (2010). A brief history of the antibiotic era: Lessons learned and challenges for the future. Front. Microbiol..

[17] Ho C.S., Wong C.T.H., Aung T.T., Lakshminarayan R., Metha J.S., Rauz S., McNally A., Kintses B., Peacock S.J., de la Fuente-Nunez C. (2024). Antimicrobial resistance: A concise update. Lancet Microbe.

[18] Perry J., Waglechner N., Wright G. (2016). The Prehistory of Antibiotic Resistance. Cold Spring Harb. Perspect. Med..

[19] Meagher K.M. (2024). Why We Should Reexamine the “Golden Age” of Antibiotics in Social Context. AMA J. Ethics.

[20] McGuinness W.A., Malachowa N., DeLeo F.R. (2017). Vancomycin Resistance in Staphylococcus aureus. Yale J. Biol. Med..

[21] Bhatt S., Chatterjee S. (2022). Fluoroquinolone antibiotics: Occurrence, mode of action, resistance, environmental detection, and remediation—A comprehensive review. Environ. Pollut..

[22] Swann M.M. (1969). Report of the Joint Committee on the use of Antibiotics. Animal Husbandry and Veterinary Medicine.

[23] World Health Organization (WHO) (2015). Global Action Plan on Antimicrobial Resistance.

[24] World Organization for Animal Health (WOAH) Terrestrial Animal Health Code. https://www.woah.org/en/what-we-do/standards/codes-and-manuals/.

[25] United Nations General Assembly (UNGA) (2016). Political Declaration of the High-Level Meeting of the General Assembly on Antimicrobial Resistance. High-Level Plenary Meeting on Antimicrobial Resistance.

[26] The European Parliament and The Council of the European Union (2019). Regulation (EU) 2019/4 of the European Parliament and of the Council of 11 December 2018 on the Manufacture, Placing on the Market and Use of Medicated Feed, Amending Regulation (EC) No 183/2005 of the European Parliament and of the Council and Repealing Council Directive 90/167/EEC. O.J. L 4. https://eur-lex.europa.eu/eli/reg/2019/4/oj/eng.

[27] European Commission (2017). A European One Health Action Plan against Antimicrobial Resistance (AMR). https://health.ec.europa.eu/system/files/2020-01/amr_2017_action-plan_0.pdf.

[28] European Commission (2020). Farm to Fork Strategy, for a Fair, Healthy and Environmentally-Friendly Food System. https://food.ec.europa.eu/document/download/472acca8-7f7b-4171-98b0-ed76720d68d3_en?filename=f2f_action-plan_2020_strategy-info_en.pdf.

[29] European Food Safety Authority (EFSA), European Centre for Disease Prevention and Control (ECDC) (2025). The European Union Summary Report on Antimicrobial Resistance in Zoonotic and Indicator Bacteria from Humans, Animals and Food in 2022–2023. EFSA J..

[30] Butaye P., Devriese L.A., Haesebrouck F. (2003). Antimicrobial growth promoters used in animal feed: Effects of less well known antibiotics on gram-positive bacteria. Clin. Microbiol. Rev..

[31] Kirchhelle C. (2018). Swann Song: Antibiotic Regulation in British Livestock Production (1953–2006). Bull. Hist. Med..

[32] Council of European Union (2003). Regulation (EC) No 1831/2003 of the European Parliament and of the Council of 22 September 2003 on Additives for Use in Animal Nutrition. O.J. L 268. https://eur-lex.europa.eu/legal-content/EN/TXT/?uri=CELEX%3A02003R1831-20210327.

[33] Sneeringer S., Bowman M., Clancy M. (2019). The U.S. and EU Animal Pharmaceutical Industries in the Age of Antibiotic Resistance. Dep. Agric. Econ. Res. Serv..

[34] World Organisation for Animal Health (WOAH) OIE Annual Report on Antimicrobial Agents Intended for Use in Animals. https://www.oie.int/app/uploads/2021/05/a-fifth-annual-report-amr.pdf.

[35] (2024). Decreto Legislativo 7 Dicembre 2023, n. 218. Adeguamento Della Normativa Nazionale Alle Disposizioni del Regolamento (UE) 2019/6 del Parlamento Europeo e del Consiglio dell’11 Dicembre 2018 Relativo ai Medicinali Veterinari e Che Abroga la Direttiva 2001/82/CE, ai Sensi Dell’articolo 17 Della Legge 4 Agosto 2022, n. 127. (23G00225). O.J. 2. https://www.gazzettaufficiale.it/eli/gu/2024/01/03/2/sg/pdf.

[36] World Health Organization (WHO) (2024). WHO List of Medically Important Antimicrobials: A Risk Management Tool for Mitigating Antimicrobial Resistance Due to Non-Human Use.

[37] European Medicine Agency: Infographic on Categorization of Antimicrobials. https://fve.org/ema-infographics-on-categorization-of-antimicrobials/.

[38] European Commission (2022). Commission Implementing Regulation (EU) 2022/1255 of 19 July 2022 Designating Antimicrobials or Groups of Antimicrobials Reserved for Treatment of Certain Infections in Humans, in Accordance with REGULATION (EU) 2019/6 of the European Parliament and of the Council. O.J. L 191. http://data.europa.eu/eli/reg_impl/2022/1255/oj.

[39] Bozzo G., Corrente M., Testa G., Casalino G., Dimuccio M.M., Circella E., Brescia N., Barrasso R., Celentano F.E. (2021). Animal Welfare, Health and the Fight against Climate Change: One Solution for Global Objectives. Agriculture.

[40] European Medicine Agency (EMA) Safety and Residues: Pharmaceuticals. https://www.ema.europa.eu/en/veterinary-regulatory-overview/research-development-veterinary-medicines/scientific-guidelines-veterinary-medicines/safety-residues-guidelines/safety-residues-pharmaceuticals.

[41] The European Parliament and The Council of The European Union (2001). Directive 2001/82/EC of The European Parliament and of the Council of 6 November 2001 on the Community Code Relating to Veterinary Medicinal Products. O.J. L 311. https://eur-lex.europa.eu/eli/dir/2001/82/oj.

[42] The European Union (1990). Council Regulation (EEC) No 2377/90 of 26 June 1990 Laying Down a COMMUNITY Procedure for the Establishment of Maximum Residue Limits of Veterinary Medicinal Products in Foodstuffs of Animal Origin. O.J. L 224. https://eur-lex.europa.eu/legal-content/EN/TXT/?uri=CELEX%3A01990R2377-20080816.

[43] Ministero Della Salute—Direzione Generale Della Sanità Animale e dei Farmaci Veterinari. Sistema Informativo Nazionale Della Farmacosorveglianza. Ricetta Veterinaria Elettronica. https://www.ricettaveterinariaelettronica.it/.

[44] Chirollo C., Nocera F.P., Piantedosi D., Fatone G., Della Valle G., De Martino L., Cortese L. (2021). Data on before and after the Traceability System of Veterinary Antimicrobial Prescriptions in Small Animals at the University Veterinary Teaching Hospital of Naples. Animals.

[45] Agenzia Italiana del Farmaco Farmacovigilanza. https://www.aifa.gov.it/farmacovigilanza1.

[46] European Medicine Agency (EMA) Pharmacovigilance (Veterinary Medicines). https://www.ema.europa.eu/en/veterinary-regulatory-overview/post-authorisation-veterinary-medicines/pharmacovigilance-veterinary-medicines.

[47] Agenzia Italiana del Farmaco (AIFA) Accesso Precoce al Farmaco e Uso Off-Label. https://www.aifa.gov.it/accesso-precoce-uso-off-label.

[48] Federazione Nazionale Ordini Veterinari Italiani (FNOVI) Farmacosorveglianza: Ruolo Primario per il Medico Veterinario. https://www.fnovi.it/sites/default/files/Dossier%20farmacosorveglianza%209-7-2012er%281%29.pdf.

[49] Local Health Authority “City of Turin” Piedmont Region Pharmacosurveillance and Pharmacovigilance. https://www.aslcittaditorino.it/farmacosorveglianza-e-farmacovigilanza/.

[50] Ventura G., Lorenzi V., Mazza F., Clemente G.A., Iacomino C., Bertocchi L., Fusi F. (2021). Best Farming Practices for the Welfare of Dairy Cows, Heifers and Calves. Animals.

[51] The Classyfarm System. https://www.classyfarm.it/index.php/vet-aziendale-it.

[52] Ministero Della Salute (2018). Decreto 7 Dicembre 2017. Sistema di Reti di Epidemio-Sorveglianza, Compiti, Responsabilità e Requisiti Professionali del Veterinario Aziendale (18A00687). O.J. 29. https://www.gazzettaufficiale.it/eli/id/2018/02/05/18A00687/sg.

[53] (2022). Decreto Legislativo 5 Agosto 2022, n. 136. Attuazione Dell’articolo 14, Comma 2, Lettere a), b), e), f), h), i), l), n), o) e p), Della Legge 22 Aprile 2021, n. 53 per Adeguare e Raccordare la Normativa Nazionale in Materia di Prevenzione e Controllo Delle Malattie Animali Che Sono Trasmissibili Agli Animali o All’uomo, Alle Disposizioni Del Regolamento (UE) 2016/429 Del Parlamento Europeo e Del Consiglio, Del 9 Marzo 2016. (22G00144) O.J. 213. https://www.normattiva.it/uri-res/N2Ls?urn:nir:stato:decreto.legislativo:2022-08-05;136.

[54] Ministero Delle Politiche Agricole Alimentari e Forestali (2022). Decreto 2 Agosto 2022 Disciplina Del «Sistema di qualità nazionale per il benessere animale». (22A06772). O.J. 279. https://www.gazzettaufficiale.it/eli/id/2022/11/29/22A06772/sg.

[55] Holighaus L., Zanon T., Kemper N., Gauly M. (2023). First evaluation of the practicability of the ClassyFarm welfare assessment protocol in Italian small-scale mountain dairy farms—A case study. Ital. J. Anim. Sci..

[56] European Parliament European Parliament Resolution of 26 November 2015 on a New Animal Welfare Strategy for 2016–2020. https://oeil.secure.europarl.europa.eu/oeil/en/procedure-file?reference=2015/2957(RSP).

[57] (2022). Gruppo di Lavoro per il Coordinamento Della Strategia Nazionale di Contrasto Dell’antimicrobico-Resistenza (GTC-AMR). Piano Nazionale di Contrasto all’Antibiotico-Resistenza (PNCAR) 2022–2025. https://www.salute.gov.it/imgs/C_17_pubblicazioni_3294_allegato.pdf.

[58] The Danish Interated Antimicrobial Resistance Monitoring and Research Programme. https://www.danmap.org.

[59] Finnish Food Authority Monitoring of Antibiotic Resistance. https://www.ruokavirasto.fi/en/animals/animal-medication/monitoring-of-antibiotic-resistance/.

[60] Frimodt-Møller N. (2004). Microbial Threat—The Copenhagen Recommendations initiative of the EU. J. Vet. Med. B Infect. Dis. Vet. Public Health.

[61] Glavind A.S., Kruse A.B., Nielsen L.R., Stege L. (2022). Monitoring antimicrobial usage in companion animals: Exploring the use of the Danish VetStat database. Acta Vet. Scand..

[62] Stege H., Bager F., Jacobsen E., Thougaard A. (2003). VETSTAT-the Danish system for surveillance of the veterinary use of drugs for production animals. Prev. Vet. Med..

[63] Ghidini S., De Luca S., Rinaldi E., Zanardi E., Ianieri A., Guadagno F., Alborali G.L., Meemken D., Conter M., Varrà M.O. (2023). Comparing Visual-Only and Visual-Palpation Post-Mortem Lung Scoring Systems in Slaughtering Pigs. Animals.

[64] Mariottini F., Giuliotti L., Gracci M., Benvenuti M.N., Salari F., Arzilli L., Martini M., Roncoroni C., Brajon G. (2022). The ClassyFarm System in Tuscan Beef Cattle Farms and the Association between Animal Welfare Level and Productive Performance. Animals.

[65] European Union (2016). Regulation (EU) No 2016/429 of the European Parliament and of the Council of 9 March 2016 on Transmissible Animal Diseases and Amending and Repealing Certain Acts in the Area of Animal Health (‘Animal Health Law’) O.J. L 84. https://eur-lex.europa.eu/legal-content/EN/TXT/?uri=CELEX%3A32016R0429.

[66] European Union (2017). European Regulation (EU) 2017/625 of the European Parliament and of the Council of 15 March 2017 on Official Controls and Other Official Activities Performed to Ensure the Application of Food and Feed Law, Rules on Animal Health and Welfare, Plant Health and Plant Protection Products, Amending Regulations (EC) No 999/2001, (EC) No 396/2005, (EC) No 1069/2009, (EC) No 1107/2009, (EU) No 1151/2012, (EU) No 652/2014, (EU) 2016/429 and (EU) 2016/2031 of the European Parliament and of the Council, Council Regulations (EC) No 1/2005 and (EC) No 1099/2009 and Council Directives 98/58/EC, 1999/74/EC, 2007/43/EC, 2008/119/EC and 2008/120/EC, and repealing Regulations (EC) No 854/2004 and (EC) No 882/2004 of the European Parliament and of the Council, Council Directives 89/608/EEC, 89/662/EEC, 90/425/EEC, 91/496/EEC, 96/23/EC, 96/93/EC and 97/78/EC and Council Decision 92/438/EEC (Official Controls Regulation). O.J. L95. https://eur-lex.europa.eu/legal-content/EN/TXT/?uri=CELEX%3A02017R0625-20250105.

[67] Woolhouse M., Ward M., van Bunnik B., Farrar J. (2015). Antimicrobial resistance in humans, livestock and the wider environment. Philos. Trans. R. Soc. Lond. B Biol. Sci..

[68] Singer R.S., Porter L.J., Thomson D.U., Gage M., Beaudoin A., Wishnie J.K. (2019). Raising Animals Without Antibiotics: U.S. Producer and Veterinarian Experiences and Opinions. Front. Vet. Sci..

[69] European Medicines Agency (EMA) (2022). Sales of Veterinary Antimicrobial Agents in 31 European Countries in 2021 Trends from 2010 to 2021 Twelfth ESVAC Report (EMA/795956/2022).

[70] Bozzo G., Barrasso R., Grimaldi C.A., Tantillo G., Roma R. (2019). Consumer attitudes towards animal welfare and their willingness to pay. Vet. Ital..

[71] Bozzo G., Dimuccio M.M. (2023). Implementation of Animal Welfare: Pros and Cons. Agriculture.

[72] Rollin B. (2001). Ethics, science and antimicrobial resistance. J. Agric. Environ. Ethics..

[73] Holman D.B., McAllister T.A., Topp E., Wright A.D., Alexander T.W. (2015). The nasopharyngeal microbiota of feedlot cattle that develop bovine respiratory disease. Vet. Microbiol..

[74] Hollis A., Maybarduk P. (2015). Antibiotic Resistance Is a Tragedy of the Commons That Necessitates Global Cooperation. J. Law Med. Ethics.

[75] Dadgostar P. (2019). Antimicrobial Resistance: Implications and Costs. Infect. Drug Resist..

[76] Jamrozik E., Heriot G.S. (2022). Ethics and antibiotic resistance. Br. Med. Bull..

[77] Waseem H., Williams M.R., Jameel S., Hashsham S.A. (2018). Antimicrobial Resistance in the Environment. Water Environ. Res..

